# Cardiac Oxidative Stress and the Therapeutic Approaches to the Intake of Antioxidant Supplements and Physical Activity

**DOI:** 10.3390/nu13103483

**Published:** 2021-09-30

**Authors:** Kosar Valaei, Shima Taherkhani, Hamid Arazi, Katsuhiko Suzuki

**Affiliations:** 1 Department of Exercise Physiology, Faculty of Sport Sciences, University of Guilan, Rasht 4199843653, Iran; kosar.valaei94@gmail.com (K.V.); shimataherkhani@msc.guilan.ac.ir (S.T.); 2 Faculty of Sport Sciences, Waseda University, 2-579-15 Mikajima, Tokorozawa 359-1192, Japan; katsu.suzu@waseda.jp

**Keywords:** reactive oxygen species, cardiovascular oxidative stress, exercise, antioxidant supplementation

## Abstract

Reactive oxygen species (ROS) are strongly reactive chemical entities that include oxygen regulated by enzymatic and non-enzymatic antioxidant defense mechanisms. ROS contribute significantly to cell homeostasis in the heart by regulating cell proliferation, differentiation, and excitation-contraction coupling. When ROS generation surpasses the ability of the antioxidant defense mechanisms to buffer them, oxidative stress develops, resulting in cellular and molecular disorders and eventually in heart failure. Oxidative stress is a critical factor in developing hypoxia- and ischemia-reperfusion-related cardiovascular disorders. This article aimed to discuss the role of oxidative stress in the pathophysiology of cardiac diseases such as hypertension and endothelial dysfunction. This review focuses on the various clinical events and oxidative stress associated with cardiovascular pathophysiology, highlighting the benefits of new experimental treatments such as creatine supplementation, omega-3 fatty acids, microRNAs, and antioxidant supplements in addition to physical exercise

## 1. Introduction

Reactive oxygen species (ROS) are cellular metabolic byproducts that are biologically produced and cause oxidative stress [1]. In addition, ROS contain oxygen that is regulated by antioxidant defense systems that are both non-enzymatic and enzymatic [2,3]. ROS modulate cell growth, and heart excitation-contraction coupling and play an integral part in cellular processes, and are useful in modulating processes involved in the maintenance of homeostasis and diverse cellular activities at low-to-moderate levels [2,4]. Natural ROS generation via the respiratory chain of the mitochondria is implicated because ROS may indeed be physiologically useful, although, in some specific circumstances, the production of ROS can be damaging to cells [2,5].

ROS are essential second messengers that transduce intracellular signals involved in different biological processes in normal quantities [6,7]. Oxidative stress arises when excessive ROS generation surpasses the antioxidant defense systems’ buffering capability or when antioxidant enzymes are impaired [6,8]. As a result, high amounts of ROS damage lipids, proteins, DNA and lead to cellular and molecular abnormalities that eventually lead to heart failure [1,2,5,9]. There are two systems against ROS scavenging: Glutaredoxins (Grxs), superoxide dismutase (SOD), catalase (CAT), glutathione peroxidase (GPx) are enzymes, while flavonoids, beta-carotene, vitamins E (Vit E), ascorbic acid (AA), ubiquinone, carotenoids, and lipoic acid are non-enzymatic antioxidants [1,10,11].

Cardiovascular diseases associated with hypoxia, including myocardial infarction, stroke, peripheral arterial disease, and renal ischemia, are the leading causes of mortality and impairment. Hypoxia is defined as the point at which oxygen concentration becomes a limiting factor for normal cellular activities such as ATP production. Hypoxia is defined as a paradigm of reactions impacting the whole body through the integration of local responses. Cardiac hypoxia occurs when the supply of oxygen falls short of the demand. Due to the significant coronary arteriovenous disparities, the myocardium cannot significantly increase oxygen supply by increasing blood oxygen extraction. As a result, the only option to satisfy the increased oxygen requirement is to expand the blood flow. Theoretically, all known processes resulting in tissue hypoxia may be responsible for decreased heart tissue oxygen delivery. However, the much more typical problems are undoubted: [1] ischemic hypoxia (often referred to as “cardiac ischemia”), which is caused by a reduction or interruption of coronary blood flow; and [2] systemic (hypoxic) hypoxia (“cardiac hypoxia”), which is defined by a decrease in PO2 levels in arterial blood despite adequate perfusion. Ischemia generally has more severe consequences than hypoxia, including lactic acidosis from anaerobic glycolysis, decreased mitochondrial energy generation, and cell death. Oxidative stress appears to be a significant pathway in several morbid conditions characterized by cardiac damage as the primary cause.

Based on physical exercise and cardiovascular function, it is well known that, in addition to enhancing cardiorespiratory capacity (VO_2_ max) [12,13], regular exercise lowers several risk predictor indices by lowering arterial blood pressure (BP), total cholesterol (TC), and LDL-cholesterol (LDL-C); it can also raise HDL-C levels and enhance endothelial function [12,14,15]. On the other hand, strenuous exercise boosts oxygen consumption and muscle consumption when performed above a certain load or by unfit or unfamiliar individuals, resulting in increased production of ROS, oxidative stress, and oxidative damage to cell macromolecules [12]. Furthermore, oxidative damage is a common basic factor in atherosclerosis, aging, and exercise-induced oxidative stress [16].

This review aimed to investigate the role of antioxidant supplementation and physical activity in improving cardiovascular oxidative stress. The first section explains an overview of the formation of ROS and antioxidant mechanisms. The sources of ROS in the cardiovascular was addressed in the second section. The third section of the emphasis of this review is the benefits of exercise on cardiovascular oxidative stress. Additionally, in the last portion, we looked at the influence of physical exercise on endothelial dysfunction and hypertension.

## 2. The Formation of ROS and Antioxidant Systems

### 2.1. Reactive Oxygen Species 

The molecules with highly reactivity produced by oxygen metabolism are known as ROS. Free radicals and non-radicals are both possible. Chemically explosive and close to the end molecules with at least one unbound valence electron at their outer shell [17,18], such as superoxide (O_2_^−^) and hydroxyl radical (OH^−^), likewise molecules like hydrogen peroxide (H_2_O_2_), may be transformed to radicals and produce hydroxyl radicals through the Fenton reaction [19]. O_2_^−^ may produce additional ROS such as H_2_O_2_ and OH^−^, and combine with nitric oxide (NO) to generate peroxynitrite (ONOO^−^) [20]. Furthermore, the Harber-Weiss reaction can produce OH^−^ by exchanging electrons between O_2_^−^ and H_2_O_2_^−^ Furthermore, when O_2_^−^ and NO are produced within several cell diameters of each other, they might spontaneously come together to form peroxynitrite (ONOO^−^) through a diffusion-limited process [21].

Normal cardiac physiology and heart function regulation, such as coronary vasodilation, platelet and neutrophil adhesion and activation inhibition, and cardiac contractile function modulation, all need nitric oxide (NO) [7,16]. Similarly, NO also protects the heart against ischemia and failure. The activation of soluble guanylyl cyclase causes a drop in intracellular Ca^2^^+^ concentration and the suppression of oxidative stress that play a part in this protective effect [21]. As a result, cytotoxic effects of O^2−^ are mediated not only by O_2_^−^ but also by the inactivation of cytoprotective NO and the production of highly reactive oxidant ONOO^−^, which is generated when NO interacts with O_2_^−^ [22]. Thus, increased ROS production causes vascular oxidative damage, which results in vascular disorder mechanisms [23]. In addition, the imbalancebetween the high activity of pro-oxidative intracellular enzymes (such as xanthine oxidase, NADPH oxidase, or the respiratory chain of the mitochondria) and low activity of anti-oxidative enzymes (such as heme oxygenase, SOD, GPx, and CAT) resulting in oxidative stress [24]. Increased ROS production causes decreased NO bioactivity, resulting in the production of the toxic ONOO^−^. Endothelial NO synthase can be “uncoupled” by ONOO^−^, leading to dysfunction of the O_2_^−^-producing enzyme, resulting in vascular oxidative stress [23,25].

### 2.2. Antioxidant Systems

Antioxidants, whether enzymes or non-enzymatic substances, inhibit the generation of free radicals and strive to repair or neutralize the harm caused by them. Several internal and external antioxidants are used to defend against oxidative injury and severe illnesses. These defenses depend primarily on antioxidants. Maintaining a careful equilibrium between oxidants and antioxidants protects healthy organisms from the damaging effects of ROS. Therefore, the constant creation of free radicals in aerobic organisms must be counterbalanced by an equivalent rate of antioxidant intake [2,26,27].

As mentioned above, several enzymes prevent the production of free radicals; some work directly to scavenge ROS (primary enzymes), while others support other endogenous antioxidants (secondary enzymes) [28].

#### 2.2.1. Enzymatic Antioxidants

##### Superoxide Dismutase

SOD is a metalloenzyme, which transforms O_2_^−^ into H_2_O_2_. The other enzymatic antioxidant systems (catalase and glutathione peroxidase) can then eliminate the H_2_O_2_ [1,29]. SODs are divided into four categories based on the metal cofactors they include. Copper-zinc SOD is found in the highest concentrations in the cytosol, chloroplasts, and extracellular space. Manganese SODs are mitochondrial, whereas iron SODs are found in plant microbial cells and cytosol [30,31]. SOD is also important in preventing ONOO^−^ production by blocking the oxidative inactivation of NO [32]. In oxidative stress-induced disease, therapeutically increasing SOD levels might be a significant therapy approach. Exogenous SODs delivery, on the other hand, might be troublesome; hypersensitivity, a short half-life, and poor absorption are all disadvantages of SOD treatment [31].

##### Catalase

CAT seems to be another antioxidant enzyme that catalyzes the hydrolysis of H_2_O_2_ to oxygen and water as a tetrameric enzyme [29,30]. CAT is widely distributed in the peroxisomes of colonic epithelium, cytoplasm, and lamina propria when the concentrations of H_2_O_2_ rise, most notably during an inflammatory reaction [31]. Based on sequence and structure, catalases are classified into three groups: pseudo catalase or Mn-catalase, catalase-peroxidase, and monofunctional catalase or typical catalase [2,32].

##### Glutathione Peroxidase 

The cytosolic enzyme GPx catalyzes the dismutation of H_2_O_2_ into oxygen and water and even the conversion of peroxide radicals to alcohols and oxygen [33]. To date, there are eight different isoforms of GPx [1,2,3,4,5,6,7,8], with GPx-1 being the most common isoform found in the cytoplasm of all mammalian cells. Glutathione peroxidase 1, a ONOO^−^ reductase enzyme prevalent throughout most cells, such as the endothelium, converts H_2_O_2_ to water and lipid peroxides to their corresponding alcohols using glutathione [27,34]. It not only scavenges H_2_O_2_ but also avoid the growth of even more hazardous radicals including OH^−^. Catalase has a lower affinity for H_2_O_2_ than GPx. It is also present in large amounts in the heart, notably in the cytosolic and mitochondrial compartments [35]. These reports have demonstrated the GPx’s principal significance as a heart protection mechanism. Furthermore, GPx is predicted to defend against oxidative damage better than SOD because increased dismutation of O_2_^−^ ions by SOD may result in a rise in H_2_O_2_ [33,34].

Whenever the production of ROS surpasses the antioxidant defense capability, oxidative stress compromises the biological tissue’s functional and structural integrity. Excessive ROS in the heart can cause myocardial remodelling, including contractile dysfunction and structural abnormalities [21].

#### 2.2.2. Non-Enzymatic Antioxidants

Chemical molecules with low molecular weight can also function directly as antioxidants. Their function is not catalytic in this circumstance. They must constantly regenerate antioxidants or obtain them from the food [2]. Non-enzymatic antioxidants may be categorized as endogenous or exogenous. Endogenous antioxidants can be synthesized by the eukaryotic cells; exogenous antioxidants must be consumed via the food.

##### Endogenous Non-Enzymatic Antioxidants

Glutathione

GSH is an essential non-enzymatic intracellular antioxidant. It is a water-soluble tripeptide with a thiol group generated from cysteine. In the cytoplasm, nucleus, and mitochondria, the reduced form of GSH is abundant [28]. It has been shown that it is the major soluble antioxidant in each cell compartment [35]. GSH production is an endergonic (ATP hydrolysis is required) reaction that begins with the condensation of glutamate and cysteine to produce -glutamylcysteine (reaction mediated by glutamate-cysteine ligase) [36]. It is protected from the action of common peptidases by this γ-peptidic bond. GSH synthetase adds a glycine residue to the -amino group of cysteine in the last step [36].

CoEnzyme Q-10 (CoQ10)

CoQ10 or ubiquinone is an isoprenoid antioxidant molecule that is also a member of the respiratory chain of the mitochondria, involved in the respiration of aerobic cells [37]. It functions as a liposoluble chain-breaking antioxidant for cell membranes and lipoproteins and a key part of energy metabolism [38]. CoQ10 exists as a redox pair at the cellular level, which means it exists in two states—ubiquinone, the oxidized form, and ubiquinol, the reduced form—that alternate constantly when CoQ10 transports hydrogen through the mitochondrial electron transfer chain. Therefore, taking ubiquinone or ubiquinol as a supplement makes no difference [39]. It protects lipoproteins and lipids from radical chain reactions, peroxidation, and oxidative stress as a few liposoluble antioxidants [40]. In addition, CoQ10, in its active form (quinol), may scavenge several ROS and regenerate other damaged antioxidants (includingVit C and E) [2].

##### Exogenous Non-Enzymatic Antioxidants

Exogenous antioxidants should be continually supplemented through the food because their synthesis routes are often found only in microbial or plant cells. Polyphenols, extracellular antioxidants found primarily on fruits and vegetables, are predominantly present in plants [41]. Moreover, carotenoids and vitamins as antioxidant micronutrients are abundant in fruits and vegetables and have been shown to improve the body’s protection against ROS and unfavorable inflammation. Several epidemiologic studies have shown that a high intake of carotenoids and vitamins with higher serum concentrations reduces the risk of cardiovascular disease [42]. Various molecular processes might explain, at least in part, the beneficial effect of polyphenols, including flavonoids, on cardiovascular health. Flavonoids, in particular, have antiplatelet, antioxidant, and anti-inflammatory activities and endothelial cell–modulating abilities. In addition, the bioactivity of flavonoids includes processes such as vascularization decrease and vasodilation stimulation. Finally, flavonoids have been demonstrated to affect key cardiometabolic risk variables such as body weight, lipid profile, blood glucose, blood pressure, and metabolic syndrome [43].

Vitamin C

Vit C is an antioxidant and a water-soluble vitamin as a radical scavenger. It protects cellular components from oxidative damage produced by ROS and free radicals. Vitamin C is a lactone (C_6_H_8_O_6_) that is available in two forms, reduced (ascorbic acid) and in the form of an oxidized (dehydro-l-ascorbic acid) [44,45], which is synthesized from glucose [35]. Studies showed that ascorbic acid could increase nitric oxide production in human’s cells of the endothelium, a system that protects arteries against myogenic tone changes (vasoconstriction), atherosclerosis, and coagulation problems [44]. The relationship between vit C consumption and coronary heart disease (CHD) risk is still debated, with different associations depending on the source of vit C [41]. In cardio-metabolic illnesses, extended endoplasmic reticulum stress and mitochondria-derived. Ascorbic acid is deprotonated at physiological pH in the reduced form of vit C. (Thus, ascorbate is found in its anion form). AA can undergo two-electron oxidation due to its high electron-donating power, yielding dehydroascorbic acid (DHA) [2]. Vit C insufficiency has been linked to various health issues, including hypertension HTN, endothelial dysfunction, cardiovascular disease, atherosclerosis, and stroke. Numerous preclinical investigations have been conducted to determine vit C’s role in cardiac and vascular protection and the amelioration of pathological illnesses [42].

Vitamin E

Vit E is a fat-soluble vitamin found in abundance in various plant oils, almonds, broccoli, and salmon. Eight different forms have been identified (α-tocopherol, β-tocopherol, γ-tocopherol, δ-tocopherol), but α-tocopherol is the most potent antioxidant, especially in cell membranes [43].

## 3. Sources of ROS in the Heart

In the heart, potential ROS producers include the mitochondrial respiratory chain, the xanthetie oxidase, decreased oxidases of nicotinamide adenine dinucleotide (NADPH), lipoxygenase, cytochrome P-450s, the nitric adenine phosphate, and peroxidases [1]. The three myocardial cell types include all these enzyme systems: cardiac myocytes, fibroblasts, and endothelial cells. While their precise proportional contributions to ROS production are not understood, mitochondria, XO, and NADPH oxidase are the primary producers of ROS, which may contribute to the pathophysiology of heart failure [46].

### 3.1. Mitochondrial Respiratory Chain ROS

Mitochondria is a significant generator of intracellular ROS. ROS generation in mitochondria is related to the partial reduction of O_2_ to O_2_^−^ by complexes I and III of the electron transfor chain (ETC) [46,47]. The premature leak of a tiny percentage of electrons to oxygen in the ETC results in the formation of physiological ROS. With mitochondria antioxidants such as SOD-2 and glutathione, the reactivity is reduced quickly by degradation or sequester O_2_^−^. The decreased mitochondrial antioxidant capability may result in heart malfunction due to many mitochondria in the cardiac tissue [48]. Mitochondrial injury or malfunction thus produces oxidative stress in mitochondrial cells [47].

Moreover, apart from the respiratory chain, it has been demonstrated that a variety of additional mitochondrial-localized proteins contributes to the mitochondrial ROS pool. P66shc, MAOs, and NOX4 are proteins. P66shc belongs to the cytosolic adaptor protein family (Src homology two domain and collagen homology region). In contrast to its p52Shc and p46Shc molecular relatives, which control Ras, p66shc played a crucial part in oxidative stress signals. The p66shc, a cytosolic protein partly locating the mitochondrial region of the membrane, helps generate ROS by oxidizing cytochrome c and promoting H_2_O_2_ production [49,50,51].

### 3.2. Xanthine Oxidase 

The XO is a xanthine oxidoreductase enzyme that produces ROS, accelerates hypoxanthine oxidation to xanthine and thereby catalyzes xanthine to uric acid oxidation [7]. It usually exists as a dehydrogenase (XDH) enzyme, but under inflammatory circumstances, the cysteine residues 535 and 992 and proteolytic conversion transition from the reductase to the oxidase (XO) form [52]. The enzyme does not only circulate in vascular and endothelial cells but also in the plasma. NADPH oxidase regulates the activity of the vascular XO as H_2_O_2_ production based on NADPH increases the activity of xanthine oxidase [53]. In conditions associated with endothelial dysfunction, vascular XO-derived ROS may be especially significant [54].

### 3.3. Nitric Oxide Synthases (NOSs)

Nitric oxide synthases (NOSs) are the enzymes in which NO and citrulline are produced as substrates from oxygen and L-arginine [55]. In this way, electrons are transported to the heme iron and BH4 in the N-terminal oxygenase domain, from NADPH attached to the C-terminal reductase domain. Three NOS isoforms are essential for the myocardium: endothelial NOS (eNOS or NOS3), inducible NOS (iNOS or NOS2), and neuronal (nNOS or NOS1) [1,56]. L-arginine, and molecular oxygen, and NADPH are co-substrates for all isoforms of NOS [56]. NO generated by eNOS is a crucial factor in vascular homeostasis. However, in some instances, eNOS may produce O_2_^−^ in place of NO, a so-called “uncoupling”, under the restricted availabilities of substrates and cofactors. Furthermore, NO may react to O_2_^−^, thus producing ONOO^−^, another strong oxidant. Excessive ONOO^−^ production causes protein nitration and leads to malfunction and death of mitochondrial and endothelial cells [1,7].

### 3.4. Nicotinamide Adenine Dinucleotide Phosphate Oxidase

NADPH oxidase refers to enzymes that catalyze electrons’ transfer by utilizing NADPH as the donor of electrons to O_2,_ producing O_2_^−^ or H_2_O_2_. The enzyme has two membrane sub-units: NOX and p22phox. The enzymes include a small GTP-binding protein Rac. The enzyme’s catalytic component NOX has many isoforms, including the first phagocytes to identify NOX2 (gp91phox) [57]. In the failing heart, NOX activity increases [58]. Interestingly, other sources may promote further ROS production from NOX. For instance, O_2_^−^ may activate XOR from NOX and BH4 degradation, as shown in diabetes and hypertension, leading to NOS uncoupling [53].

## 4. MiRNA

Microribonucleic acids (MicroRNAs) comprise 22 naturally occurring nucleotides that control gene expression by annealing to specific messenger RNAs, preventing translation, or increasing messenger RNA (mRNA) degradation. Several microRNAs, notably miR-1, are muscle-specific among the 2000 microRNAs discovered in humans. MiR-1 accounts for 40% of all miRNAs, according to deep sequencing of miRNAs from cardiac tissue. Given that both ROS generation and microRNA (miRNA) transcription signatures have been linked to the development of CVDs, it is critical to understand the ROS-miRNA interaction. Several studies have found that miRNAs play critical roles in cardiovascular growth, pathology, regeneration, and repair and that they may be utilized to diagnose and treat cardiovascular disorders such as hypertrophy, myocardial infarction, contractility abnormalities, and arrhythmias. Additionally, many miRNAs have been recognized as moderators of oxidative stress in the cardiovascular system through their regulation of ROS producers, antioxidant signaling pathways, and specific antioxidant mechanisms [59].

Growing data has indicated that miRNAs may be regarded as possible targets and stimulators of oxidative-stress-related pathways [60]. As mentioned before, oxidative stress is a critical factor in developing various cardiovascular disorders, including hypoxia, ischemia/reperfusion damage, and heart failure [61]. While intracellular ROS are generated generally due to normal mitochondrial respiration, they are also generated during reperfusion in hypoxic tissue and connection with infection and inflammation, resulting in pathological cardiac diseases [62]. One of the consequences of ROS generation in cardiomyocytes is a change in noncoding RNA (ncRNA) expression, which contributes to cell death and heart disease. MiRNAs are the most extensively studied of these ncRNAs since they significantly affect heart disease by limiting protein expression or mRNA degradation [63].

## 5. Omega-3

Omega-3 fatty acids (FAs) have been widely researched in animal models and vitro, controlled nutrition studies, epidemiology researches, and randomized controlled trials (RCTs) in humans [64]. Omega-3 is an appealing preventative approach owing to its potential to decrease cardiac cell sensitivity to oxidative stress damage. This impact is achieved via various processes, including enhanced antioxidant defenses, changes in membrane fluidity, and the capacity to block intracellular calcium from being released in response to oxidative stress [65].

According to experimental data, the antioxidant effects of omega-3 are linked to their absorption into cell membranes and regulation of redox signaling pathways. In rats, omega-3 supplementation enhances antioxidant enzymes’ expression and function while also lowering the levels of thiobarbituric acid-reactive compounds (TBARS). Oxidized omega-3 interacts directly with Keap1, a negative moderator of Nrf2, causing Keap1 to dissociate from Cullin3 and activating Nrf2-dependent antioxidant genes such heme oxygenase-1. Supplementation with omega-3 is linked to a decrease in myocyte sensitivity to ROS-induced IR damage, as well as an elevation in SOD and GSH-Px production [66]. Animal experiments have shown that PUFA has cardioprotective benefits via up-regulating heat shock protein 72, a critical preconditioning protein, and increasing the omega-3 component of cardiac membranes, which seems to aid the defensive response to hypoxic damage. Compared to non-treated rats, hearts supplemented with omega-3 exhibited smaller infarcts and greater left ventricular pressure. Compared with the non-supplemented group, hearts in the omega-3 supplemented group had lower oxidative stress indicators, more significant antioxidant activity, decreased function, and NF-B and Nrf2 activation [61]. Experimental studies in vitro and in vivo indicate that omega-3 fatty acids reduce heart rate and blood pressure directly by regulating the activity of numerous ion channels and maintaining cardiomyocyte membranes, indirectly by enhancing left ventricular diastolic filling and boosting vagal tone, or both [67]. Additionally, these FAs enhance endothelial function by increasing the translocation and activation of endothelial nitric oxide synthase into the cytosol, resulting in vasodilation [68].

As a major risk factor for cardiovascular disease, endothelial dysfunction is caused by increased pro-inflammatory cytokines and adhesion molecules, decreased nitric oxide synthesis, and altered plasminogen activator inhibitor-1 balance [69]. Omega-3 fatty acids in the diet may enhance endothelial function by modifying endothelial cell membrane fluidity and composition, increasing vessel relaxation and constriction, and reducing adhesion molecules and inflammatory cytokines production [70]. Wang et al. [71] showed that omega-3 fatty acids improved NO bioavailability in patients with CVD but not in healthy individuals over an average of 56 days at doses varying from 0.45 to 4.50 g/day. One other study examining the effects of dietary omega-3 fatty acids on hyperlipidemic, hypertensive, and diabetic populations found that increasing omega-3 fatty acid intake for a year resulted in a decrease in vascular cell adhesion molecule 1 (VCAM-1) and intercellular adhesion molecule 1 (ICAM-1) levels, which was associated with an improvement in endothelial function in peripheral small arteriolar arteries [72]. Omega-3’s positive effects on cardiac function may potentially result in a reduction in blood pressure. Indeed, 12 weeks of dietary FO treatment decreased blood pressure and enhanced endothelial function in spontaneously hypertensive rats, and these benefits were linked with inhibition of sphingolipid-dependent cardiovascular activation [73]. Additionally, many clinical studies indicated that eiocosapentaenoic acid (EPA) and docsahexaenoic acid (DHA) had distinct hemodynamic effects. For instance, Mori et al. [74] randomized overweight males with hyperlipidemia to receive 4 g/day of either EPA, DHA, or olive oil (as a control group) during a 6-week period. Patients receiving DHA had a statistically significant reduction in blood pressure and heart rate, while those getting EPA did not [74]. These results provide a biological and clinical foundation for understanding how omega-3 fatty acids benefit the vascular system by modifying endothelial dysfunction and hypertension.

## 6. Creatine Supplementation

Creatine is a natural compound that plays a critical role in supplying cellular energy and intracellular energy in combination with creatine kinase. It is produced endogenously and is found exogenously in various dietary sources, including meats and fish [75]. Recent data suggest that creatine supplementation has a variety of positive effects on various cellular functions that are not energy-related. Among these promising benefits is creatine’s antioxidant capacity, which scavenges and neutralizes ROS implicated in various diseases [76]. There are many mechanisms in which creatine may be therapeutically helpful to endothelial function. For example, creatine may help minimize oxidative stress, promote the antioxidant system, and lower circulating homocysteine levels and chronic or acute inflammation [77]. Santacruz et al. [78] shown that creatine supplementation enhanced the phosphocreatine level of in vitro cardiomyocytes. Notably, these authors observed a similar rise under hypoxic circumstances, even though hypoxia reduced creatine absorption. On another one, Matthews et al. [79] were among the first to explore the ability of creatine to defend against oxidative stress and neurotoxicity produced by intrastriatal malonate or intraperitoneal nitropropionic acid (3-NP) injections, both of which are used as Huntington’s disease animal models. The existence of a CRT and the capacity of creatine supplementation to enhance brain reserves of energy metabolites were first shown after two weeks of 1% dietary creatine supplementation. Furthermore, the scientists found that rats given creatine for two weeks had substantially fewer striatal lesions after malonate or 3-NP neurotoxicity, suggesting considerable neuroprotection. Finally, Matthews found that creatine inhibited the production of hydroxyl (OH) free radicals. These results prompted the first hypothesis that creatine has antioxidant-like characteristics. Despite the increasing amount of research on creatine’s antioxidant capabilities, relatively few studies have examined creatine’s potential to show these same characteristics in humans. Rahimi et al. [80] examined the impact of creatine supplementation (4 × 5 g/day for 7 days) on exercise-induced oxidative stress after a resistance exercise session. This double-blind, placebo-controlled research evaluated oxidative stress in twenty-seven healthy young men immediately and 24 h after exercise using plasma malondialdehyde (MDA) and urine 8-hydroxy2-deoxyguanosine (8-OHdG). Rahimi found that individuals supplemented with creatine had substantially lower levels of 8-OHdG immediately and 24 h after exercise than those who received a placebo. Additionally, the placebo group’s plasma MDA concentrations were substantially greater after exercise. Rahimi stated that these findings confirmed creatine’s capacity to protect cells from oxidative damage. It was produced as a result of intense resistance training. Despite these encouraging results, more study is necessary to understand creatine as an antioxidant in people thoroughly.

Considering the majority, if not all, CVDs are accompanied by oxidative stress and consequent vascular dysfunction, supplementary creatine’s capacity to operate as an antioxidant is only one of the new ways to improve vascular health [81]. Given that oxidative stress can impair NO synthesis, function, and bioavailability, it is reasonable to hypothesize that if creatine possesses antioxidant properties, it may aid in reducing and scavenging ROS, thereby increasing NO bioavailability contributing to improved vascular health [81].

## 7. Exercise and Stress Oxidative in the Heart

Many factors that cause cell homeostasis loss, including physical activity, often damage the heart muscle, while exercise has been recommended for health reasons [82,83]. Exercise-induced changes in blood flow, arterial dilation, or vasodilatory responses can provide information about the cardiovascular function and endothelial dysfunction that is not visible at rest. In exercise-induced vasodilation, NO plays a key role [84], and higher free radicals and oxidative stress can also cause reductions in exercise-induced vasodilation because decreased vasodilation might reduce perfusion during exercise [85]. It is important to figure out how to increase vasodilation with exercise. It is also unclear how antioxidant supplementation would alter genetic variations in exercise or the possibility of increased exercise vasodilation and blood flow [84,85]. Exhausting activities, extended workouts, overtraining syndrome, and overcoming limitations as a phase of overtraining syndrome’s early beginnings cause a significant oxidative stress response. Moderate exercise, low-intensity training, and long-term training, on the other hand, boost endogenous antioxidant status [2]. Together with lifestyle adjustments, physical activity is thought to be the most effective non-pharmacological treatment for various chronic conditions, particularly cardiovascular disorders [2].

Regular exercise has been shown to positively affect the progression of cardiovascular disease, as it increases antioxidant capacity and reduces oxidative stress, which leads to cellular homeostasis and balance of oxidation. Regular physical activity boosts the expression of key antioxidant enzymes, decreases pro-oxidant ones, enhances the total antioxidant capacity, and improves MDA levels in the heart induced by oxidative damage [86]. Based on the hormesis theory, low to moderate oxidative stress produced by exercise promotes beneficial exercise-related physiological changes. Additionally, prolonged exposure to oxidative damage caused by physical exercise may result in several hormesis adaptations, such as activating antioxidative defense systems [87]. Podgorska et al. [88] investigated the impact of aerobic training on athletes’ endothelium profile and platelet function. Their findings indicated that no variations in the amounts of NO pathway metabolites existed. The control group had greater PAI-1 levels after ASA therapy and sICAM-1 levels at the beginning and following ASA, but no changes in MDA, 6-keto-PGF-1 alpha, or platelet clumping were observed. Thus, it seems that regular physical activity affects endothelial function but not platelet activation, suggesting that it may have an impact on total heart disease risk. Regular exercise induces transitory increases in ROSproduction that elicit adaptive responses rather than deleterious consequences and activates signal transduction pathways that promote positive adaptations [89].

### 7.1. Endothelial Dysfunction, Cardiac Oxidative Stress and Exercise

The vascular endothelium, which controls the flow of molecules and circulating cells from the blood to the tissues, is a primary target of oxidative stress. Endothelial dysfunction contributes to the pathophysiology of various heart illnesses and disorders such as heart failure, where the changed redox state increases ROS production, which is mainly regulated by rising oxidative stress and NO bioavailability changes [90]. It seems that an inconsistency between NO bioavailability and ROS leads to oxidative damage, which is a mark of cardiac disease [91]. Due to strong vasodilatory, anti-inflammatory and antithrombotic properties of NO, it plays a primary role in endogenous antioxidant protection [92]. The majority of vascular NO is produced by eNOS, a cytochrome p450 reductase-like enzyme that converts L-arginine to NO tetrahydrobiopterin. Thus, reduced NO bioavailability is mainly caused by increased NO degradation induced by ROS, decreased eNOS production, substrate or cofactor shortage for eNOS, and an improper activation of eNOS caused by insufficient cellular signalling [93]. Moreover, uncoupling eNOS leads to decreased bioavailability of NO when eNOS switches its enzymatic activity to produce O_2_^−^ and H_2_O_2_ instead of NO [94].

In metabolic and cardiovascular disorders, the vascular endothelium is the primary source of dysfunction [95]. NADPH is a primary oxidative stress source in the arterial wall, which has a vital role in ROS generation and NO scavenging. However, the vascular wall included several enzymes such as SOD, GPx, and CAT that serve as antioxidant defense mechanisms, reducing the ROS capacity [96,97]. Regular physical activity has various health advantages, including increasing body composition and endothelial function and helping to reduce insulin resistance, oxidative damage, and hypertension [98]. Increasing blood flow and shear stress during physical activity promotes better vascular homeostasis by decreasing ROS generation, rising NO bioavailability in the endothelium, and helping to enhance endothelial function [99]. Repeated bouts of increased blood flow during exercise result in an improvement in endothelial function, contributing to the long-term advantages of regular exercise in reducing the risk of cardiovascular disease. This process is most likely to entail a persistent increase in the generation of NO mediated by increased eNOS expression [100]. Doroszko et al. [101] investigated the role of NO metabolism mechanism in developing endothelial dysfunction within men with and without HTN. These findings showed no significant changes in NO pathway metabolites at baseline or after indomethacin therapy. In contrast, L-arginine and indomethacin showed a synergistic beneficial impact on FMD in hypertensive individuals. The authors reported that in young normotensive males, ED is mainly caused by a lack of NO. Problems in the metabolism of prostanoids have a significant influence in reducing NO bioavailability in young hypertensive males. The significant incidence of ED in otherwise healthy individuals indicates that ultrasonography FMD assessment is a critical tool for cardiovascular risk stratification.

Short-term and medium-term exercise has been shown to enhance NO-related vasodilation, while long-term exercise has been linked to arterial remodeling. Additionally, it is critical to note that while laminar shear stress caused by exercise is the primary antioxidant and boosts endothelial function, oscillatory shear stress caused by hypertension has the opposite effect, increasing oxidative damage and oxidative vasculature damage via a significant improvement in NADPH activity [102].

### 7.2. Hypertension, Cardiac Oxidative Stress and Exercise

HTN is a significant risk factor for heart diseases development, including stroke and coronary artery disease. HTN is characterized by a persistent increase of systolic and diastolic blood pressure over 140/90 mmHg and is categorized as essential hypertension [103]. Exercise is a critical element of lifestyle treatment for cardiovascular disease such as hypertension [104,105]. Physical activity can reduce high blood pressure using different mechanisms [86]. For instance, exercise-induced blood pressure reduction occurs due to beneficial changes in oxidative damage, inflammation, and endothelial function [104]. Human essential hypertension has often been shown to be linked with oxidative damage. It should be noted that a significant number of clinical investigations have shown that ROS generation is increased in hypertension. Thus, a significant reduction in ROS generation regulated with higher levels of antioxidant defense has been proposed, which is the primary mechanism through which physical activity reduces blood pressure [86]. The improvement of systolic and diastolic blood pressure and nitrate concentration is associated with a high level of physical activity. Therefore, the mechanism controlling blood pressure may include an increased antioxidant capacity that can be achieved through a high level of exercise and greater NO bioavailability. Furthermore, it has been shown that physical activity can boost NO generation and also reduce the inactivation of NO, resulting in rising NO bioavailability and the improvement of endothelial activity [103]. Regular exercise plays a crucial role in the reversibility of endothelial dysfunction in high blood pressure, which is effective and safe for treating hypertension. Active muscles have been shown to release various cytokines, and peptides collectively referred to as myokines and exhibiting anti-inflammatory activity, which improves the bioavailability of NO by reducing the generation of ROS [103].

Also, it has been shown that different types of exercise have beneficial effects on hypertension. For example, moderate-intensity exercise affects reducing blood pressure by decreasing inflammatory cells [86]. Cook et al. [106] have reported that resistance training is also efficient in regulation matrix formation proteins and oxidative damage; moreover, the results showed that this type of physical activity helped enhance exercise performance in the possible protection of hypertension’s early development [106].

**Figure 1:**
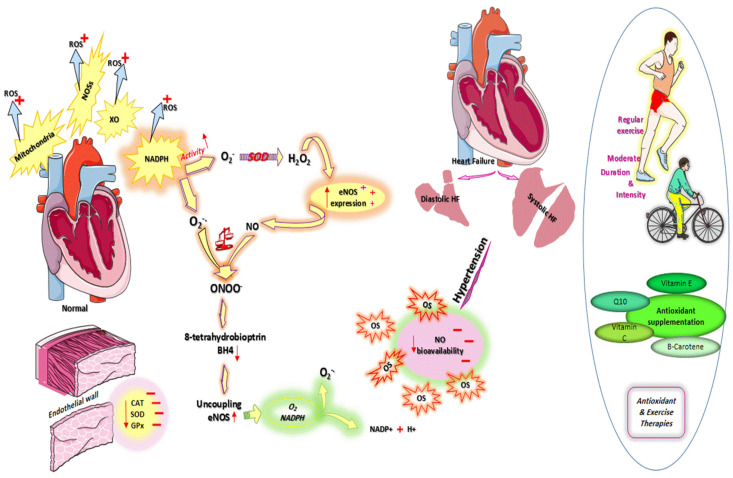
Cardiovascular oxidative stress. OS: Abbreviations: oxidative stress; ROS: reactive oxygen species; HF: heart failure; NOSs: nitric oxide synthases; XO: xanthine oxidase; NADPH: nicotinamide adenine dinucleotide phosphate hydrogen; SOD: superoxide dismutase; GPx: glutathione peroxidase; CAT: catalase; Enos:endothelial nitric oxide synthase; BH4: 8-tetrahydrobiopterin; O_2_^−^: superoxide anion; ONOO^−^: Peroxynitrite.

Additionally, aerobic exercise plays an important role in reducing the production of ROS and reducing the incidence of ROS-related diseases such as hypertension. Aerobic activity increases adaptation to oxidative stress by raising antioxidant levels. Therefore, eNOS phosphorylation is improved, and also the expression of antioxidant enzymes is increased. Due to aerobic exercise, blood pressure is reduced or enhanced due to improved bioavailability of NO, increased eNOS expression, and decreased O_2_^−^ levels [107] (Figure 1).

## 8. Antioxidant and Physical Activity Therapies for Heart Disease Related to Oxidative Stress

Various studies have investigated that in addition to physical activity, antioxidant supplements as compounds that can neutralize the activity of oxygen species and free radicals can protect against many diseases, including cardiovascular diseases such as high blood pressure, and heart failure [1]. However, some studies have not found any significant changes in the effectiveness of antioxidant interventions and exercise for cardiovascular disease and risk factors.

Michishita and colleagues looked to determine the relationships between an increased SBP reaction to exercise and food consumption in normotensive individuals. Michishta et al. invited normotensive men and women without heart disease for their study. The participant’s nutrient consumption including vitamin E, sodium and potassium was checked using a self-administered questionnaire in addition to performing graded exercise tests. It was concluded by authors, while potassium, dietary sodium/potassium ratio, dietary fiber, Vit A, B2, C, and E intake were all associated with resting SBP, after adjusting for resting SBP, the dietary sodium/potassium ratio and Vit E consumption were significantly associated with an excessive SBP response to exercise. Additionally, it was shown that the % change in SBP during exercise, the dietary Na/K ratio, and vitamin E consumption are dose-dependent correlations. Therefore, one could hypothesize that an increased SBP response to exercise was related to the dietary sodium/potassium ratio and vitamin E consumption in normotensive individuals, regardless of resting blood pressure [108]. Tropea et al. investigated the impact of 200 mg/kg/day of grape seed extract polyphenols (GSEP) on the function of the resistant arteries in pregnant eNOS mice between gestational day (GD) 10.5 and GD 18.5. Considering the association between GSEP actions and oxidative stress, these findings suggest a potential benefit of GSEP on reducing the concentration of plasma malondialdehyde (MDA) and maternal SBP concentration. On another interesting note, Tropea found that GSEP was not capable of altering vascular reactivity but capable of improving the relaxation of vascular endothelial-dependent in eNOS mice’s mesenteric and uterine arteries [109]. In an attempt to explore the impact of 500 mg of Cynara Scolymus L, twice daily for eight weeks on blood pressure (BP), Ardalani et al. [110] looked to evaluate the clinical efficacy of C.scolymus on BP and body mass index (BMI) in patients suffering from hypertension. Interestingly, it was reported that the consumption of C.scolymus powder, which had enough flavonoid and antioxidants, despite reducing BMI, could not improve SBP and DBP. These findings could be related to a low dosage of supplementation. Banday et al. [111] looked to determine the impact of BSP (a prooxidant) and tempol (T) within Sprague Dawley rats (SD) for three weeks. The subjects took the supplements at a dose of 1mM BSO and 1Mm T, respectively. Interestingly, it was shown that oxidative stress affects vascular NO-PKG-VASP signaling, resulting in a rise in blood pressure, reversing these abnormalities with antioxidant supplementation. After three weeks of treatment with BSO, Sprague Dawley rats developed oxidative stress and hypertension. In response to NO donors, the blood arteries of these rats demonstrated reduced vasorelaxation. Therefore, tempol supplementation of rats treated with BSO decreased oxidative challenge and blood pressure while restoring NO signaling. Considering the relationships between high-intensity interval training (HIIT) and moderate-intensity continuous training (MICT) and vascular resistance, YC Huang et al. [112] reported that after six weeks of HIIT and MICT for 30 min/day, findings showed that both HIIT and MICT increase the function of the RA reservoir. However, only HIIT improves the function of the RA conduit to strengthen RV preload. Thus, HIIT results in more significant chamber dilation than MICT by raising the radial strain rate during systole and diastole while reducing radial strain. While both treatments reduce RV afterload, PVR, and RVSP at rest, only HIIT reduces PVR during hypoxic exercise. Notably, the correlation analysis revealed that an increased RVEF is linked with improved RA reservoir and circuit activity and a reduced PVR after HIIT. In other hand, Omar, J.S et al. [113] investigated the impact of regular swimming exercise on metabolic syndrome risk factors in humans who have type 2 diabetes (T2DM) and hypertension (HTN). These individuals were subjected to regular swimming for 2 h, 3 times/week for 16 weeks for 6 weeks. The authors reported that statistical analysis revealed that post-test total cholesterol (TC), high-density lipoprotein (HDL), low-density lipoprotein (LDL), triglycerides (TG), BMI, body fat percent, blood glucose (BG), SBP, and DBP variables were significantly improved between groups (experimental vs. control). As a consequence of these findings, frequent swimming workouts seem to have a beneficial effect on metabolic syndrome. In patients with hypertension, Waclawovsky and colleagues [114] examined the effects of resistance exercise (RE), aerobic exercise (AE), and combined exercise (CE) on endothelial progenitor cells (EPCs), flow-mediated dilation (FMD), progenitor cells (PCs), endothelial-cell derived microvesicles (EMVs), and oxidative stress markers for one set of three types of exercise. These individuals were subjected to AE, RE, and CE. The main results of their study showed that a single session of moderate-intensity exercise did not affect brachial artery FMD. One session of moderate-intensity exercise did not affect brachial artery FMD. Moreover, in the absence of the main finding, the levels of PCs were significantly changed, resulting in decreased. Furthermore, their exercise program had no effect on EPC, oxidative stress, or EMV levels. Due to the absence of evidence of endothelial cells damage and the fact that the redox state and circulating EMV levels remained unchanged, this finding may indicate that a single session of AE, RE, or CE at times studied was safe and preserved vascular integrity in hypertensive individuals who had decreased endothelial regenerative capacity. Wray DW et al. [115] employed older mildly hypertensive men as subjects to evaluate blood pressure (BP), FMD, DBP, and SBP after 3 times/week for 6 weeks of knee-extensor exercise. They consumed 2 doses of α-lipoic acid (300 mg in both doses), vitamins C (500 mg in both doses), and vitamin E (200 and 400 I.U, respectively). Authors reported these findings that the low muscle mass exercise program resulted in a substantial decrease in resting and exercise-induced arterial blood pressure and improved endothelium-dependent FMD, demonstrating the effectiveness of this non-invasive, low-stress treatment on endothelial function. Interestingly, acute oral antioxidant treatment before training had no meaningful effect on arterial blood pressure or FMD. Interestingly, immediate antioxidant treatment restored arterial blood pressure to pre-training levels after exercise training and substantially reduced FMD. Thus, it seems that this adverse result after the integration of the two beneficial therapies highlights the complicated nature of oxidative damage in vivo, where pro- and antioxidant effects interplay to maintain normal vasomotor tone. These results indicate that, while exercise alone induces a relevant adaptation to increased oxidative stress, the acute reduction in free radical concentration following antioxidant administration may have eliminated ROS with positive vasoactive assets in the exercise-trained state. Hemati et al. [116]. found that a combination of supplemental creatine monohydrate and physical activity can decrease blood levels of inflammatory parameters such as hs-CRP or IL-6 in individuals with heart failure. At the study’s conclusion, the mean blood levels of inflammatory and endothelial dysfunction indicators were dramatically higher in the control group than in their baseline condition. On the other hand, the study’s findings indicated that exercise and creatine monohydrate successfully lowered the mean blood levels of inflammatory markers, primarily IL-6, in the intervention group. Additionally, when mean blood levels of inflammatory markers were compared between control and intervention groups, it was shown that regular exercise in conjunction with supplementary creatine monohydrate had a substantial lowering impact on these indicators in HF patients. Regular exercise and creatine monohydrate also affected blood levels of indicators associated with endothelial dysfunction. Similar to markers of inflammation serum, endothelial dysfunction markers were boosted compared to the baseline in the control group; however, the levels of serum of p-selectin and VCAM-1 were reduced, though not significantly, in the intervention group, while serum levels of ICAM-1 were significantly decreased. There was a significant difference between the intervention and control groups for all endothelial dysfunction indicators. Mitochondrial failure decreases energy generation and increases oxidative stress, altering the DNA structure, lipids, and proteins due to this occurrence. Creatine monohydrate enhances mitochondrial activity and therefore ameliorates the diseases as mentioned above (Table 1).

## 9. Conclusions

Cardiovascular disorders can arise and evolve in part as a result of oxidative stress. An increasing amount of data indicates that oxidative stress plays a critical role in developing various cardiac diseases. Numerous treatment methods have been expanded to combat oxidative stress. While some experiments have shown the positive benefits of antioxidant and exercise treatment in various heart diseases, findings in human clinical trials vary significantly. A better knowledge of oxidative stress in heart diseases and more efficient exercise in addition to antioxidant medicines are required to treat cardiovascular diseases more effectively.

## Figures and Tables

**Table 1:** Antioxidant and physical activity therapies.

Reference	Subject	Antioxidant Supplementation	Physical Activity	Result
Michishta et al. (2019) [108]	Normotensive men and women without heart disease	Vitamin E, Sodium and Potassium	Graded exercise test	↑ SBP
Tropea et al. (2020) [109]	Pregnant mice	GSEP	-	↑ resistance artery function
Ardalani et al. (2020) [110]	Hypertensive patients	E. Scolymus powder (500 mg twice daily)	-	↑ BMI and SBP
Banday et al. (2019) [111]	Male SD rats	BSO and T	-	↑ BMI and SBP
YC Huang et al. (2021) [112]	Young and healthy sedentary males	-	HIIT and MICT 30 min/day (total six weeks)	↓ pulmonary vascular resistance with HIIT
Omar, J.S et al. (2021) [113]	Both gender with HTN and T2DM	-	Regular swimming (6 weeks)	↑ HDL, LDL, SBP, and DBP
Waclawovsky, G et al. (2021) [114]	Hypertensive males	-	AE, RE and CE	↔ in oxidative stress and FMD
Wray DW et al. (2009) [115]	Older mildly hypertensive men	Vitamin E, C and α-lipoic acid	Knee-extensor exercise	↔ in BP, FMD. But exercise ↑ DBP, SBP and FMD
Dewell et al. (2011) [115]	Healthy adults with ≥one elevated risk factor	Antioxidant pills mixed (Vitamin C, E, Selenium and beta carotene)	-	↔ in inflammatory markers
Hemati et al. (2016) [116]	Heart failure patient	Creaine monohydrate (5 g/day)	8 weeks, 3 times/week Aerobic exercise (20–40 min with 60–80% heart rate)	↓ IL-6, hs-CRP p-selection, VCAM-I levels

↑ increase; ↓ decrease; ↔ no change; SBP: systolic blood pressure; GSEP: grape seed extract polyphenols; BMI: body mass index; SD: Sprague dawley; HIIT: high-intensity interval training; MICT: moderate-intensity continuous training; HTN: hypertension; T2DM: type 2 diabetes mellitus; HDL: high-density lipoprotein; LDL: low-density lipoprotein; DBP: diastolic blood pressure; AE: aerobic exercise; RE: resistance exercise; CE: combined exercise; FMD: flow-mediated dilation; CRP: C-reactive protein; VCAM-I: vascular cell adhesion molecule-1.
